# The changing microRNA landscape by color and cloudiness: a cautionary tale for nipple aspirate fluid biomarker analysis

**DOI:** 10.1007/s13402-021-00641-w

**Published:** 2021-10-16

**Authors:** Susana I. S. Patuleia, Elsken van der Wall, Carla H. van Gils, Marije F. Bakker, Agnes Jager, Marleen M. Voorhorst-Ogink, Paul J. van Diest, Cathy B. Moelans

**Affiliations:** 1 Department of Pathology, University Medical Center Utrecht, Utrecht University, Heidelberglaan 100, 3584 CX Utrecht, The Netherlands; 2 Department of Medical Oncology, University Medical Center Utrecht, Utrecht University, Utrecht, The Netherlands; 3 Department of Epidemiology of the Julius Center for Health Sciences and Primary Care, University Medical Center Utrecht, Utrecht University, Utrecht, The Netherlands; 4 Department of Medical Oncology, Erasmus MC Cancer Institute, Rotterdam, The Netherlands

**Keywords:** Breast cancer, Nipple aspirate fluid, Liquid biopsy, miRNAs, Biomarkers, NAF color, miRNA confounders, Sample features

## Abstract

**Purpose:**

Investigation of nipple aspirate fluid (NAF)-based microRNAs (miRNAs) as a potential screening tool for women at increased risk of developing breast cancer is the scope of our research. While aiming to identify discriminating NAF-miRNAs between women with different mammographic densities, we were confronted with an unexpected confounder: NAF sample appearance. Here we report and alert for the impact of NAF color and cloudiness on miRNA assessment.

**Methods:**

Seven classes of NAF colors coupled with cloudiness appearance were established. Using 173 NAF samples from 154 healthy women (19 samples were bilaterally collected), the expression of 14 target and 2 candidate endogenous control (EC) miRNAs was investigated using Taqman Advanced miRNA assays to identify significant differential expression patterns between color-cloudiness classes. Inter- and intra-individual variation of miRNA expression was analyzed using the coefficient of variation (CV).

**Results:**

We found that between the seven NAF classes, fold change miRNA expression differences ranged between 2.4 and 19.6 depending on the interrogated miRNA. Clear NAF samples exhibited higher miRNA expression levels compared to cloudy NAF samples with fold change differences ranging between 1.1 and 6.2. Inter-individual and intra-individual miRNA expression was fairly stable (CV < 15 %), but nevertheless impacted by NAF sample appearance. Within NAF classes, inter-individual variation was largest for green samples (CV 6-15 %) and smallest for bloody samples (CV 2-6 %).

**Conclusions:**

Our data indicate that NAF color and cloudiness influence miRNA expression and should, therefore, be systematically registered using an objective color classification system. Given that sample appearance is an inherent feature of NAF, these variables should be statistically controlled for in multivariate data analyses. This cautionary note and recommendations could be of value beyond the field of NAF-miRNAs, given that variability in sample color and cloudiness is likewise observed in liquid biopsies such as urine, cerebrospinal fluid and sputum, and could thereby influence the levels of miRNAs and other biomarkers.

**Supplementary Information:**

The online version contains supplementary material available at 10.1007/s13402-021-00641-w.

## Introduction

MicroRNAs (miRNAs) are key regulators in many cellular biological processes. They represent a class of short (∼22 nucleotides long), non-coding RNAs that modulate gene expression at the post-transcriptional level [1]. The miRNA repository miRBase (release 22.1) currently lists 2654 mature miRNAs in *Homo sapiens* [2], and an estimated 30–80 % of human genes are influenced by at least one of these miRNAs [3, 4]. Cellular miRNAs can be released into biofluids as a result of apoptotic or necrotic cell death or active secretion [5]. These circulating, cell-free miRNAs hold great promise as a new class of cancer biomarkers due to their surprisingly high stability in biofluids, correlation with carcinogenesis and disease state and ease of measurement [5–13]. Among the various body biofluids in which miRNAs can be measured, nipple aspirate fluid (NAF) has attracted attention for breast cancer early detection and management because it may reflect pathological changes in the breast microenvironment [14–16]. NAF is secreted in small amounts by the breast ducts of adult non-lactating women and can be collected by non-invasive vacuum aspiration preceded by oxytocin nasal spray administration [17–19]. The use of oxytocin promotes the release of already existing fluid in the milk ducts, thereby yielding sufficient material (on average 10-20 µl) for molecular analysis in the majority of healthy volunteers and patients [14, 15, 17, 20–23]. NAF collection causes less discomfort compared to other breast cancer screening modalities [14, 20], and the synchronous acquirement of matched pairs of bilateral NAF samples provides intra-patient control samples for unilateral disease.

NAF-based miRNA assessment in the context of early breast cancer detection in women at increased risk of developing breast cancer, such as those having high mammographic density, is the focus of our ongoing investigations. While searching for discriminatory miRNAs between two groups of healthy women differing in mammographic density, we identified NAF sample color and cloudiness as relevant confounders. Even though these sample features have previously been described as being associated with lifestyle factors and nutrient composition [24–26], they have never been described as having a remarkable influence on biomarker discovery. Moreover, interpretation of NAF sample appearance is prone to subjectivity and, hence, its reporting lacks consistency. The following color designations were extracted from the literature: pale-yellow, dark yellow, brown, brown-black, brown-cloudy, green-brown, light-green, greenish-clear, olive color, green, green-milky, white, white cloudy, milky, colored, colorless, gray-cloudy, black, clear, cloudy and opaque [21, 24–30]. This highlights the need for a standardized system to allow future between-study comparisons.

To elucidate the influence of the neglected NAF features color and cloudiness on miRNA biomarker assessment, we established seven classes of NAF sample appearance and studied their association with miRNA expression levels.

## Materials and methods

### Ethics, sample collection and processing

To evaluate the effect of NAF color and cloudiness on miRNA expression levels, 173 NAF samples from 154 women were included (19 samples were bilaterally acquired from the same women) from the DENSE-on biobank (biobank numbers 14-467 and 15-356). 92 of these NAF samples were collected from 82 women at screening age (50-74 years old) with extremely dense breasts according to Volpara imaging software, version 1.5 (Volpara Health Technologies) [31], i.e. with a Volpara density 4 or ‘d’; this is the highest of a 4-point radiological classification, which is comparable to a ‘d’ in the breast density categories of the Breast Imaging, Reporting and Data System (BI-RADS) of the American College of Radiology [32]. 81 samples were collected from 72 women ≤ 60 years old undergoing screening with a breast density at the other end of the spectrum, i.e., Volpara density 1 or ‘a’, which is a result of an almost entirely fatty breast tissue composition. The DENSE-on biobank was set up to gain a better biological understanding of breast density, e.g. why some women have extremely dense breasts and if there are biomarkers for early breast cancer detection in these women. The biobank is an extension of the Dutch nationwide multicenter Dense tissue and Early breast Neoplasm ScrEening (DENSE) trial (NCT01315015 [33–35]), which aims to investigate the additional value of MRI in screening for women with extremely dense breasts.

The studies were approved by the Institutional Review Boards of the participating hospitals, and the UMC Utrecht Biobank Research Ethics Committee. Written informed consent was provided by all participants. Samples were collected between June 2015 and March 2016. The median age of the participants was 55 years (interquartile range (IQR) = 6). Other anthropomorphic characteristics are listed in Supplementary Table 1. All participants were healthy at the time of NAF collection, without abnormalities on recent mammography and/or MRI. NAF samples were collected after nasal oxytocin administration and application of vacuum around the breast by a modified breast pump, as described previously [20, 21]. The collected fluid was conserved in a buffer solution (50 mM Tris pH 8.0, 150 mM NaCl, 2 mM EDTA) and, without centrifugation, immediately snap-frozen at -80 °C until analysis.

### NAF color and cloudiness categorization into NAF classes

Upon NAF collection, the research nurses assessed and registered NAF appearance on color and cloudiness. Given that 22 different NAF appearances were reported during study visits (Supplementary Table 2), a lumped classification was made based on the most prevalent NAF appearances registered to reduce the number of NAF classes for subsequent statistical analysis. The 173 NAF samples were subdivided into 7 color classes (Supplementary Table 2) coupling colors with cloudiness appearance: 53 clear-colorless samples (31 %), 36 bloody samples (including red, orange and pink; 21 %), 30 cloudy-white samples (17 %), 20 clear-yellow samples (11 %), 19 cloudy-yellow samples (11 %), 8 green (5 %) and 7 brown samples (4 %).

### RNA extraction and quantification

For all experiments, an AllPrep DNA/RNA/miRNA Universal Kit (Qiagen, Hilden, Germany) was used to extract total RNA according to the manufacturer’s protocol. Ten µl NAF was used to evaluate the effect of NAF color and cloudiness on miRNA expression levels. Non-human ath-mir-159 (with a 5’ phosphate) was spiked-in at 300 pg by pre-mixing with RLT plus lysis buffer. RNA was eluted in 30 µl RNAse-free water. The concentration of the extracted RNA was measured by Qubit 3.0 (ThermoFisher Scientific, MA, USA) fluorometric high sensitivity quantification. Next, all RNA samples were stored at -80 °C until further analysis.

### Reverse transcription, pre-amplification and Taqman Advanced miRNA analysis

To study associations between NAF color and cloudiness and miRNA expression levels, the expression of 16 human mature miRNAs was evaluated using individual Taqman Advanced miRNA assays (ThermoFisher Scientific, Catalog number A25576): hsa-miR-19a-3p, hsa-miR-25-3p, hsa-miR-29a-3p, hsa-miR-29b-3p, hsa-miR-125a-5p, hsa-miR-99b-5p, hsa-miR-155-5p, hsa-miR-181a-5p, hsa-miR-186-5p, hsa-miR-187-3p, hsa-miR-222-3p, hsa-miR-324-5p, hsa-miR-339-5p, hsa-miR-361-5p, hsa-miR-425-5p and hsa-miR-660-5p (see Supplementary Materials for assay identification numbers (IDs)). These microRNAs were evaluated because they initially demonstrated potential discriminatory power between women with Volpara 1 and Volpara 4 mammographic densities when no correction for NAF appearance had been performed.

Hsa-miR-99b-5p and hsa-miR-25-3p were used as suitable endogenous control (EC) miRNAs for NAF as they demonstrated the lowest geNorm M-values in previous miRNA profiling experiments using the same platform (low M-values indicate high expression stability) [36]. Hsa-miR-99b-5p, with the lowest M-value of the two candidate EC miRNAs, was selected as EC for differential expression analysis of target miRNAs in this study.

According to the manufacturer’s instructions, 5 ng total RNA was first poly-A tailed, and after adaptor ligation and reverse transcription, pre-amplified for 14 cycles. The pre-amplification product was subsequently diluted 10x in 0.1x TE buffer pH 8.0. qPCR was performed in duplicate in a 20 µl final volume using a Taqman Fast Advanced Mastermix (ThermoFisher Scientific) on a ViiA7 realtime PCR device. All miRNA amplification plots were visually inspected on curve shape and signal timing. Threshold cycles (CTs), i.e., the cycle at which the fluorescence level reaches a certain value (the threshold), above the threshold of 35 were omitted, as were samples with aberrant spike-in values. Next, CT values were used for calculation of the delta CT (DCT = CT(target miRNA)−CT(endogenous control miRNA)). Subsequently, the commonly used delta delta CT (DDCT) was calculated (2^−DDCT^, where DDCT = DCT(target sample)-DCT(reference sample)) [37, 38] resulting in relative fold changes in target miRNA expression between color classes.

### Statistical analysis

Statistical analyses were performed using IBM SPSS Statistics for Windows version 25.0.0.2 (IBM Corp., Orchard Road Armonk, New York, USA). Variables analyzed in relation to NAF appearance were age (continuous), body mass index (BMI, continuous), breast density (dichotomous) and miRNA expression levels (continuous). Normality of data distribution was evaluated by Kolmogorov-Smirnov test. Data are presented as median with IQR or mean with standard deviation. A *p* value < 0.05 was considered statistically significant.

MiRNA median CT differences (median CT (target miRNA class 1)-median CT (target miRNA class 2)) and subsequent fold changes (FCs) between NAF classes (clear-colorless, bloody, cloudy-white, clear-yellow, cloudy-yellow, green and brown) were calculated. Kruskall-Wallis test was used to identify miRNAs with significant differential expression (based on DDCT) between NAF classes. Mann-Whitney U test was used to identify miRNAs with significant differential expression between clear and cloudy NAF samples, and between any two NAF classes. The coefficient of variation (CV) was calculated as the ratio of the standard deviation to the mean miRNA expression (CT value) within and across all color classes, as well as intra-individually between left and right breasts. A criterion for intra-individual CV analysis was a minimum detection of 6 out of the 16 miRNAs per sample. Consequently, three NAF pairs were omitted from final intra-individual analysis. CVs below 15 % were considered to have acceptable technical reliability [39]. Unsupervised hierarchical clustering of NAF samples based on their miRNA expression pattern was performed using the web tool ClustVis [40]. Missing values were automatically imputed by ClustVis. GraphPad Prism 8.3 for Windows (San Diego, California USA) was used for graphical visualization of the results.

## Results

### NAF appearance classes are associated with age

**Fig. 1:**
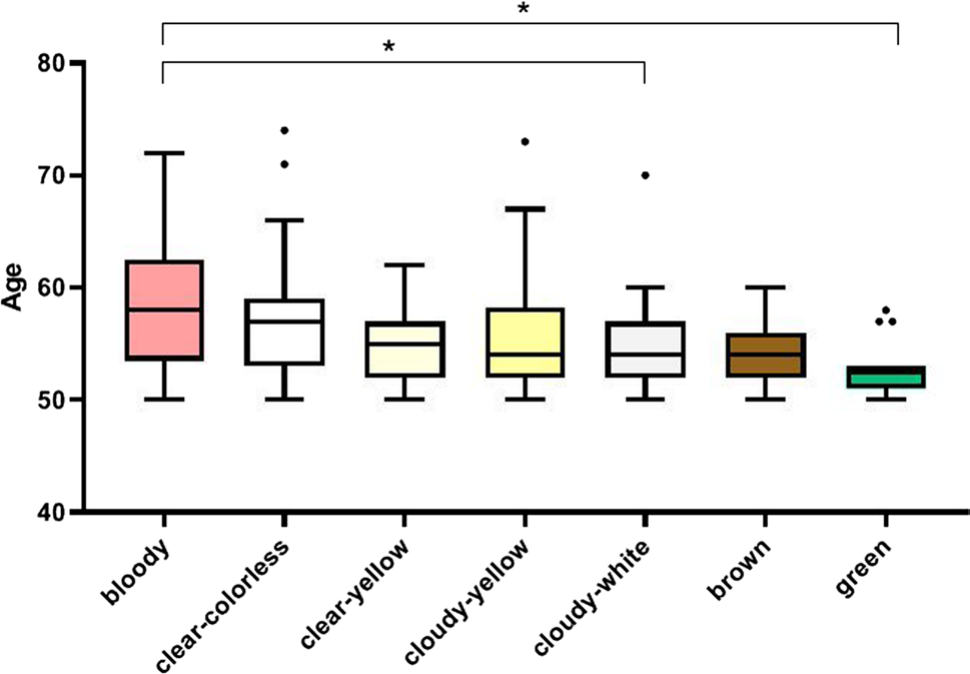
Association between nipple aspirate fluid classes and age. The most commonly occurring NAF appearance classes from older to younger age were bloody, clear-colorless, clear-yellow, cloudy-yellow, cloudy-white, brown and green. Boxes extend from the 25th to 75th percentiles (IQR). The horizontal line in the box is plotted at the median. Tukey method was used to indicate outliers (25/75th percentile ± 1.5* interquartile range). Significant differences are indicated with *

We found that NAF appearance classes were significantly associated with age (*p* = 0.001 between 7 NAF classes). Bloody NAF samples were more frequently observed in older women, especially compared to green NAF (*p* = 0.002) and cloudy-white NAF (*p* = 0.014). Overall, the order of most commonly observed NAF classes, from older to younger age (ranging between 50 and 74 years old), was as follows: bloody, clear-colorless, clear-yellow, cloudy-yellow, cloudy-white, brown and green samples (Fig. 1). No significant association between NAF appearance and BMI or breast density was noted.

### RNA concentration varies significantly with NAF color and cloudiness

Next, we found that the total RNA concentration significantly varied per NAF appearance class (*p* < 0.0001), with highest concentrations observed in bloody (n = 48; 19.9 ng/µl, IQR = 43.29) and brown (n = 14; 29.5 ng/µl, IQR = 56.79) NAF samples. This was followed by clear-yellow NAF (n = 19; 9.97 ng/µl, IQR = 19.69), clear-colorless NAF (n = 48; 6.35 ng/µl, IQR = 8.71), green NAF (n = 12; 4.02 ng/µl, IQR = 12.44), cloudy-white (n = 15; 3.01 ng/µl, IQR = 5.70) and cloudy-yellow (n = 17; 3 ng/µl, IQR = 2.99) NAF (Supplementary Fig. 1). The RNA concentration was significantly higher when it was extracted from clear compared to cloudy NAF samples (*p* < 0.0001). Median concentrations were 12 ng/µl (IQR = 26.9) and 3.85 ng/µl (IQR = 5.79) in clear NAF (n = 111) and cloudy NAF (n = 57), respectively.

### MiRNA expression varies significantly with NAF color and cloudiness

**Fig. 2:**
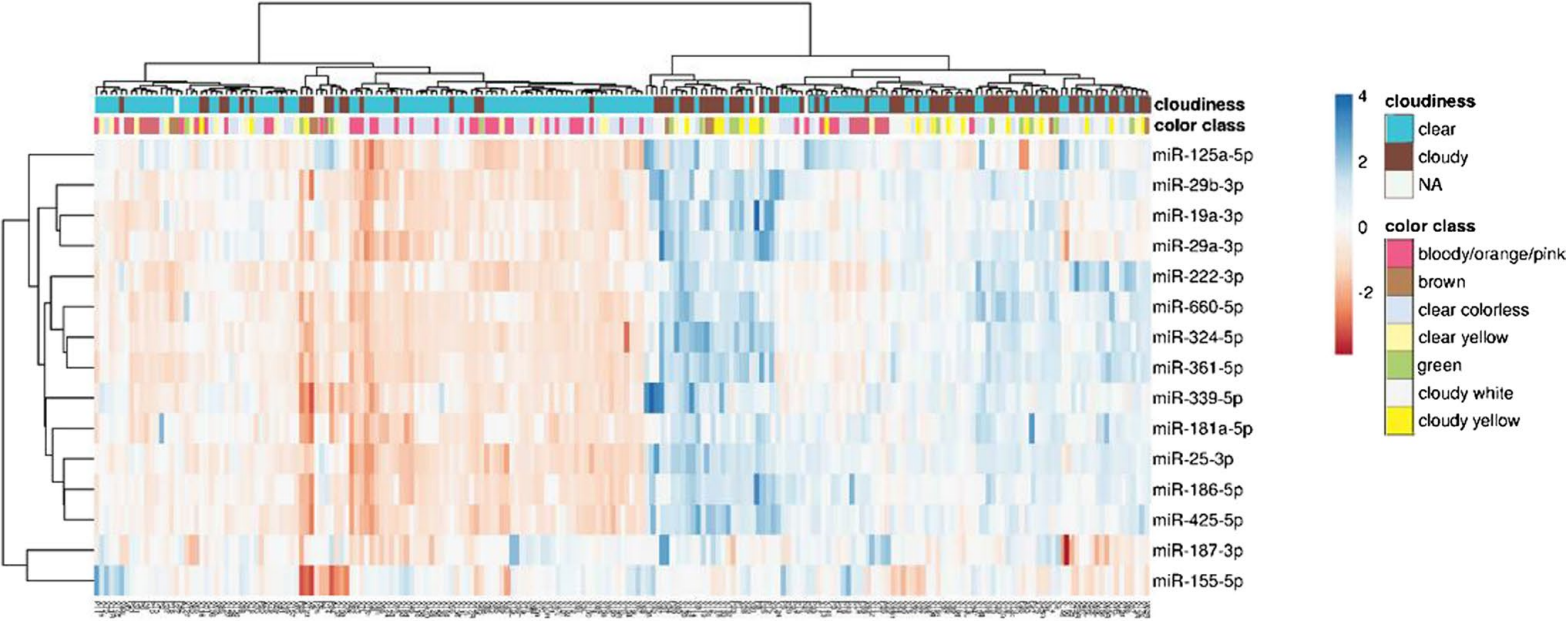
Unsupervised hierarchical clustering of 211 nipple aspirate fluid samples based on the expression pattern (delta delta CT) of 15 miRNAs. Rows are centered and unit variance scaling is applied to rows. Imputation was used for missing value estimation. Both rows and columns are clustered using Euclidean distance and Ward linkage

Of the 15 interrogated target miRNAs, all but one (hsa-miR-187-3p) were significantly differentially expressed between NAF colors (all miRNAs *p* < 0.0001, except hsa-miR-125-5p with *p* = 0.008) and NAF cloudiness (all miRNAs *p* < 0.0001 except hsa-miR-125-5p with *p* = 0.036). In general, clear NAF samples showed higher miRNA expression levels compared to cloudy NAF samples with median CT (expression) differences between cloudy and clear samples ranging from 0.16 for hsa-miR-125a-5p (FC = 1.12) to 2.63 for hsa-miR-222-3p (FC = 6.19) (Supplementary Table 3A). Interestingly, we found that hsa-miR-155-5p was the only miRNA with a higher expression in cloudy versus clear NAF (FC = 3.81). Between the seven NAF classes, median CT differences ranged from 1.26 for hsa-miR-187-3p (FC = 2.39) to 4.29 for hsa-miR-222-3p (FC = 19.64) despite identical RNA input based on Qubit total RNA measurements. Combinatorial analyses among the seven NAF classes were also performed, leading to a total of 21 class comparisons. In general, cloudy-white, cloudy-yellow and green NAF showed lower miRNA expression levels compared to red, brown and clear-colorless NAF. In contrast, hsa-miR-155-5p showed a higher expression in green samples compared to clear-colorless and clear-yellow samples (Supplementary Table 3B). In almost every NAF class comparison, at least one miRNA showed a significantly different pattern. Exceptions were green, cloudy-white and cloudy-yellow NAF, exhibiting no significantly different miRNAs between NAF classes. Brown and clear-yellow NAF were also highly similar for all interrogated miRNAs. Greatest differences were observed between bloody and green NAF, between bloody and cloudy-yellow or cloudy-white NAF and between clear-colorless and cloudy-yellow or cloudy-white NAF, with at least 13/15 interrogated miRNAs showing significantly different expression levels. Accordingly, unsupervised hierarchical clustering of NAF samples based on their 15-miRNA expression pattern resulted in two major clusters, one containing mainly cloudy NAF samples (white, yellow, green or brown), and the other containing mainly clear NAF samples (Fig. 2). Within the latter cluster, clear-colorless and bloody samples were separated from clear-yellow and brown samples.

### Candidate endogenous control miRNA choice significantly impacts variability between NAF color and cloudiness classes

**Fig. 3:**
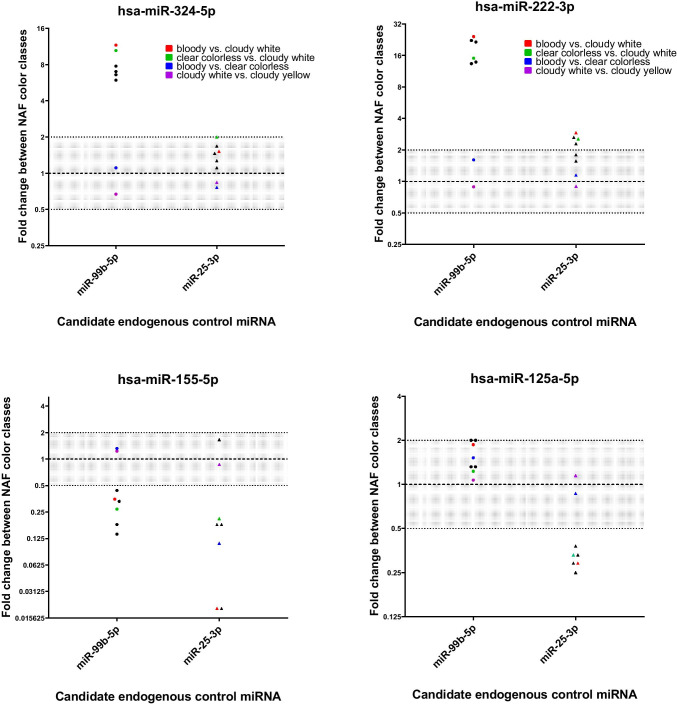
The effect of endogenous control miRNA choice on variability between nipple aspirate fluid (NAF) color-cloudiness classes. Fold changes between NAF color classes (derived from median DDCT) are plotted for 4 target miRNAs (hsa-miR-324-5p, hsa-miR-222-3p, hsa-miR-155-5p and hsa-miR-125a-5p) based on two endogenous controls (hsa-miR-99b-5p and hsa-miR-25-3p). Fold changes between 0.5 and 2 are shaded grey. The upper two target miRNAs show less variability between color classes when using hsa-miR-25-3p as endogenous control, the lower two miRNAs show less variability when using hsa-miR-99b-5p as endogenous control. Overall, variability between bloody and clear-colorless samples (blue), and between cloudy-white and cloudy-yellow samples (purple) was acceptable, regardless of the chosen endogenous control. Variability was larger between other color classes (e.g. colors red = bloody versus cloudy-white, and green = clear-colorless versus cloudy-white)

Since miRNA expression is influenced significantly by NAF color, choosing the right endogenous miRNA may prove to be difficult. Figure 3 illustrates the effect of candidate endogenous control choice on between-sample variability in the context of color. Overall, variability between bloody and clear-colorless samples, and between cloudy-white and cloudy-yellow samples was acceptable, regardless of the chosen endogenous control. Variability was, however, much larger between other NAF classes (e.g. bloody or clear-colorless versus cloudy-white). For hsa-miR-324-5p, for instance, a miRNA with significantly lower CT values (higher non-normalized expression) in bloody and clear-colorless versus cloudy-white and cloudy-yellow NAF, after normalization with hsa-miR-99b-5p (DDCT) showed median FCs between 11.6 and 7.0 depending on the comparison (red and colorless vs. cloudy-white and cloudy-yellow). However, when normalized against hsa-miR-25-3p, showing an effect of similar magnitude as observed for hsa-miR-324-5p, median FCs varied between 1.27 and 2.0. As hsa-miR-155-5p seems to show an opposite effect compared to the other interrogated miRNAs (higher CT values and thus lower expression in bloody and colorless samples), a suitable endogenous control miRNA for proper normalization is yet to be determined.

### Inter-individual and intra-individual microRNA expression variation in the context of NAF color

**Fig. 4:**
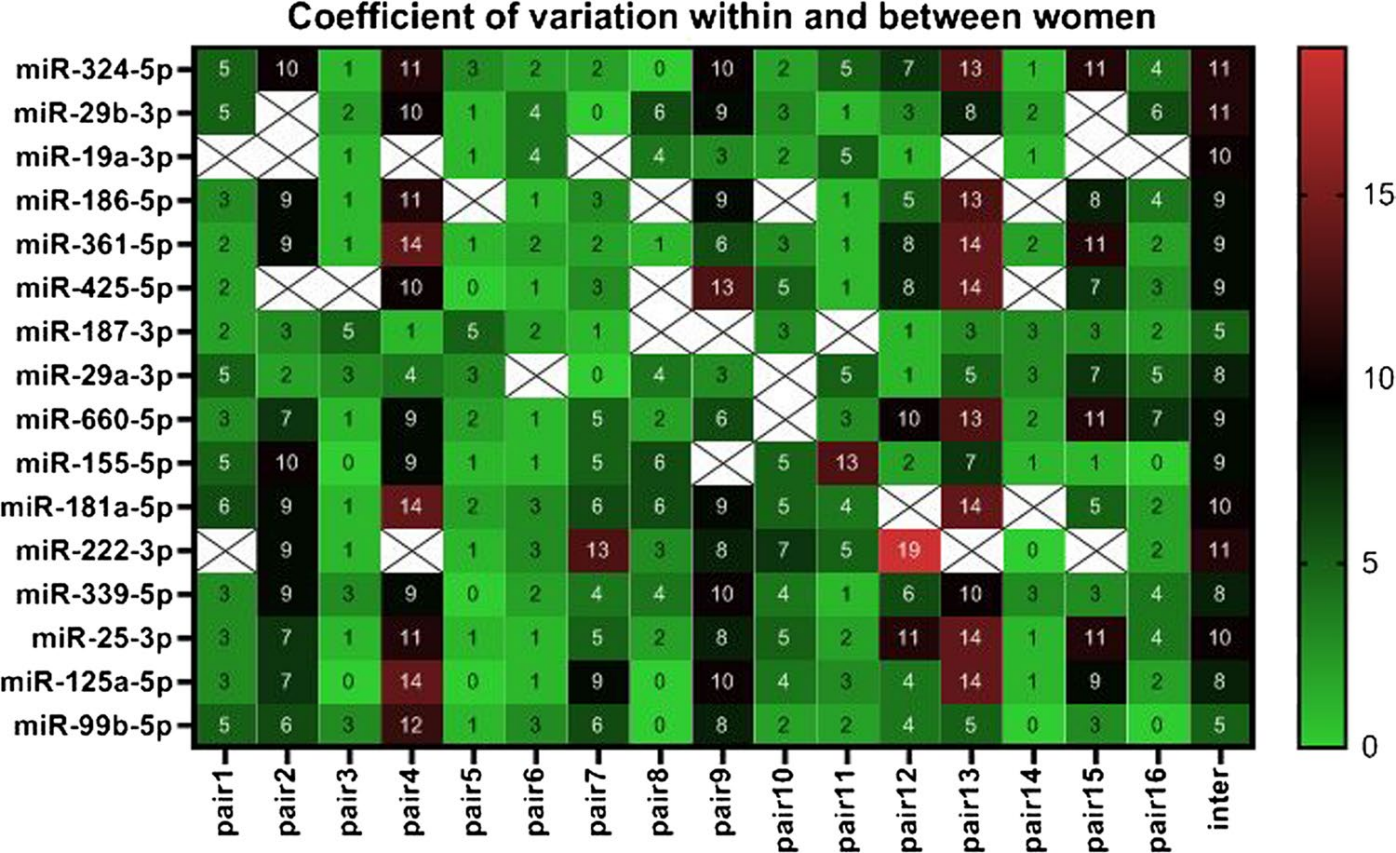
Heatmap of coefficient of variation (CV) for nipple aspirate fluid miRNA expression within and between women. CV was based on mean CT values and standard deviations determined for 16 miRNAs in 135 women (inter-individual variation; 1 breast) and an additional 16 women (intra-individual variation; left and right breast pairs). Samples with reliable measurements for at least 6 of the 16 interrogated miRNAs (15 miRNAs and EC hsa-mir-99b-5p) were selected for this figure. Crossed boxes are missing values for the miRNAs

Inter-individual coefficients of variation (CV) across all NAF classes ranged between 5 % for hsa-miR-187-5p and hsa-miR-99b-5p, and 11 % for hsa-miR-222-3p, hsa-miR-324-5p and hsa-miR-29b-3p. Within NAF classes, inter-individual variation was largest for green samples (CV 6-15 %) and smallest for bloody samples (CV 2-6 %). Intra-individual differences between left and right breast, based on 16 pairs of samples, were of similar magnitude, with smallest CV for hsa-miR-187-5p and hsa-miR-19a-3p (both 1-5 %), and largest CV for hsa-miR-222-3p (0.3-19 %). Of the 16 pairs of samples, 7 pairs had the same NAF appearance while 9 pairs had different NAF appearances. Inter-breast differences were generally larger with differing NAF color. For example, hsa-miR-361-5p and hsa-miR-425-5p tended to show notable inter-breast differences with differing NAF color (*p* = 0.053 and *p* = 0.085, respectively) while no significant difference was observed when the NAF color class was identical between breasts (*p* = 0.341 and *p* = 0.384, respectively). Figure 4 depicts a heatmap of CV percentages for NAF miRNA expression levels within and between women. In general, using hsa-miR-99b-5p as endogenous control, the maximal absolute DDCT difference between left and right breasts varied between 2.1 (hsa-miR-339-5p) and 6.1 (hsa-miR-222-3p). Maximal absolute DDCT differences between individual women varied between 5.5 (hsa-miR-125a-5p) and 14.3 (hsa-miR-155-5p). Using hsa-miR-25-3p as endogenous control, the maximal absolute DDCT difference between left and right breasts varied between 1.3 (hsa-miR-324-5p) and 5.0 (hsa-miR-222-3p). Maximal absolute inter-individual DDCT differences varied between 4.6 (hsa-miR-186-5p) and 12.5 (hsa-miR-155-5p).

## Discussion

MicroRNAs are considered promising disease biomarkers given their renowned stability in liquid biopsies and their expression levels reflecting subtle changes in pathological state [5–11]. Before they can make the step from bench to bedside, their longitudinal, intra-subject and inter-subject stability should be explored in independent cohorts. Alongside, factors that may cause intrinsic noise in miRNA expression, such as study subject characteristics, pre-analytic procedures or sample characteristics must be identified and controlled for. Many such factors have been identified in blood samples [41–43], but are yet unexplored in NAF. NAF can present itself with a variety of colors in combination with a cloudy or clear appearance. Even though liquid sample appearance is quite relevant for clinical assessment (e.g. urine, sputum, drain fluids or cerebrospinal fluid), such a variable is scarcely reported in the evaluation of new biomarkers in liquid biopsies [44–53] and has seldom been investigated with the purpose of evaluating its intrinsic influence on (mi)RNA levels. Here, we present the association of NAF sample appearance with individual characteristics, and evaluate its effect on intra-individual and inter-individual miRNA expression variability and normalization.

We found that NAF color-cloudiness is associated with woman’s age. Overall, red, clear-colorless and clear-yellow NAF were found to be more common in older women. The only other study comparing NAF color-cloudiness and age showed that older age increased the chance of lighter NAF colors [26] which is in line with our findings. However, that study used other color classes and a broader age range (20-70 years old compared to 50-74 years old in our study). Moreover, we show that NAF color and cloudiness significantly influence total RNA concentrations and miRNA expression levels. A more specific analysis stratifying NAF for color and cloudiness showed that the effect of the former seems to be somewhat secondary to the effect of the latter. Unsupervised cluster analysis clearly separated cloudy from clear NAF samples based on miRNA expression patterns, but subsequent sub-clustering into NAF colors was less distinct, except for the highly similar clear-colorless and bloody NAF specimens.

Intra-individual and inter-individual miRNA expression variability were overall below the CV threshold of 15 %, thereby showing acceptable miRNA stability in NAF samples within and amongst study subjects. As expected, variability of miRNA expression levels was greater inter-individually than intra-individually. Specifically for intra-individual variability, a sub-analysis showed that this was somewhat greater between samples from different color categories compared to those of the same color category. Sample appearance not only influenced inter-individual and intra-individual miRNA expression variation, but also posed a problem for normalization, as candidate endogenous control miRNAs might not vary with cloudiness and color to the same extent as target miRNAs. We therefore recommend using a combination of endogenous control miRNAs and to correct for sample characteristics in multivariable analysis.

Identifying the underlying cause of these different NAF colors in healthy women may explain our findings. We hypothesize that several potential factors may contribute to NAF color such as diverse cellular [54] or bacterial [55] compositions in NAF samples, nutrients and proteins [56–58], medications [59–62] or food intake [62–68]. But studies addressing the association between these possible factors and color classes are limited. Only two studies by Petrakis et al. from over three decades ago reported an association between NAF colors and nutrients [24, 25]. In those studies, NAF samples of darker colors (dark yellow, brown, green and black) were shown to have a higher concentration of lipids, cholesterol, estrogens, NA^+^ and K^+^ and a lower lactose concentration compared to NAF samples of lighter colors. A comparison between our data and those of Petrakis et al. is hampered by the different color classes used and the fact that we did not obtain the nutrient information studied by Petrakis et al. for our cohorts.

A more recent and meticulous study on NAF composition per sample color is lacking. We attempted to identify NAF cell types per NAF class using fluorescence-activated cell sorting (FACS). However, this was hampered by sample viscosity, the sparse volume and the presence or very limited numbers of cells in the NAF samples, a finding also reported by others [63, 69–75]. Single cell sequencing, a technique that allows analyzing samples with a small number of cells, may help to decompose the cellular composition of NAF per color class, but this technique still needs to be tested in this context.

Cellular composition is important as several studies have shown that blood cells, especially red blood cells, white blood cells and platelets, are major contributors to cell-free miRNAs [76–80]. This has resulted in the introduction of a routine quality control step for serum and plasma miRNA analysis [81, 82], consisting of calculating the ratio of hsa-miR-451a (enriched for in red blood cells) to hsa-miR-23a-3p (not affected by hemolysis). Since bloody was one of our NAF classes, we hypothesized that those samples would contain more red blood cells, in line with the fact that these samples have the highest RNA concentration and a high miRNA expression. We found, however, that the suggested ratio for serum and plasma could not be extrapolated to NAF because of a different physiological expression of both miRNAs in this particular biofluid. NAF samples that were visually red, orange or pink did not show CT differences > 5 (indicating hemolysis by the abovementioned ratio) but rather < 0, with no clear differences when compared with non-red samples (data not shown). Another method for the identification of red blood cell contamination is measuring oxy-hemoglobin absorbance by spectrophotometry [77, 83]. Again, usage of this technique was hampered by NAF sample viscosity and cloudiness.

The analysis of a restricted number of miRNAs, namely 15 or 5 miRNAs, can be considered a limitation of the present study. An investigation of a broader range of miRNAs using a non-targeted or multi-targeted approach, such as profiling or sequencing, may allow the identification of miRNAs most susceptible to NAF appearance. Another limitation of the present study is that color and cloudiness are subjective variables. Future research with a standardized, objectified color scale, such as the international RAL (which stands for ‘*ReichsAusschuss für Lieferbedingungen*’ in German) [84] or Pantone [85] systems for color categories, should be performed to allow uniformity of data reporting and, hence, data comparison between future studies. As an alternative to wallpaper matching of NAF colors with RAL colors, image analysis techniques or, even better, artificial intelligence techniques could be employed. The latter approach, however, requires large sample sizes to properly train convolutional neural networks. As a consequence of the accidental findings here reported, we have already started implementing the RAL system in our NAF studies (Supplementary Fig. 2) and a study investigating discriminatory miRNAs between density classes, correcting for NAF color classes.

## Conclusions

In conclusion, we show that NAF sample color and cloudiness may serve as relevant variables that should be systematically registered by adopting an objective color classification system such as RAL, and that may to be taken into consideration in biomarker analyses. We recommend using a combination of endogenous control miRNAs and to correct for sample characteristics in statistical multivariable analyses. Our cautionary note and recommendations could be of value beyond the field of NAF-miRNAs, given that sample color variability is also seen in other liquid biopsies such as urine, cerebrospinal fluid and sputum, and could influence miRNA and other biomarker levels, thereby hampering biomarker discovery and validation.

## Supplementary Information

ESM 1 (DOCX 14.9 KB)

ESM 2 (DOCX 75.9 KB)

ESM 3 (DOCX 15.7 KB)

ESM 4 (XLSX 175 KB)

## Data Availability

The authors confirm that the data supporting the findings of this study are available within the article and its supplementary files.

## Abbreviations

NAF: nipple aspirate fluid
microRNAs: miRNAs
EC: endogenous control
CV: coefficient of variation
DENSE: Dense tissue and Early breast Neoplasm ScrEening
IQR: interquartile range
CT: threshold cycles
DCT: delta CT
DDCT: delta delta CT
FC: fold changes
BMI: body mass index
RAL: ReichsAusschuss für Lieferbedingungen
