# Supplementary material for: The changing microRNA landscape by color and cloudiness: a cautionary tale for nipple aspirate fluid biomarker analysis
Source: Cell Oncol (Dordr). 2021 Oct 16;44(6):1339–49. doi: 10.1007/s13402-021-00641-w (PMC8648697; doi:10.1007/s13402-021-00641-w)
Supplement: Supplementary file 1 — (DOCX 14.9 KB) [file 13402_2021_641_MOESM1_ESM.docx]

**Supplementary Materials.** Assay identification numbers (IDs) of the human mature microRNA used.

| **Human mature microRNA** | **Assay ID** |
| --- | --- |
| **hsa-miR-16-5p** | 477860_mir |
| **hsa-miR-19a-3p** | 479228_mir |
| **hsa-miR-21-5p** | 477975_mir |
| **hsa-miR-25-3p** | 477994_mir |
| **hsa-miR-29a-3p** | 478587_mir |
| **hsa-miR-29b-3p** | 478369_mir |
| **hsa-miR-99b-5p** | 478343_mir |
| **hsa-miR-125a-5p** | 477884_mir |
| **hsa-miR-155-5p** | 483064_mir |
| **hsa-miR-181a-5p** | 477857_mir |
| **hsa-miR-186-5p** | 477940_mir |
| **hsa-miR-187-3p** | 477941_mir |
| **hsa-miR-200c-3p** | 478351_mir |
| **hsa-miR-222-3p** | 477982_mir |
| **hsa-miR-324-5p** | 483066_mir |
| **hsa-miR-339-5p** | 478040_mir |
| **hsa-miR-361-5p** | 478056_mir |
| **hsa-miR-425-5p** | 478094_mir |
| **hsa-miR-660-5p** | 478192_mir |
