# Supplementary material for: The changing microRNA landscape by color and cloudiness: a cautionary tale for nipple aspirate fluid biomarker analysis
Source: Cell Oncol (Dordr). 2021 Oct 16;44(6):1339–49. doi: 10.1007/s13402-021-00641-w (PMC8648697; doi:10.1007/s13402-021-00641-w)
Supplement: Supplementary file 2 — (DOCX 75.9 KB) [file 13402_2021_641_MOESM2_ESM.docx]

**Supplementary Figure 1.** Association between nipple aspirate fluid appearance classes and total RNA concentration.

**Supplementary Figure 2**. Selection of RAL colors to register upon nipple aspirate fluid collection (1).


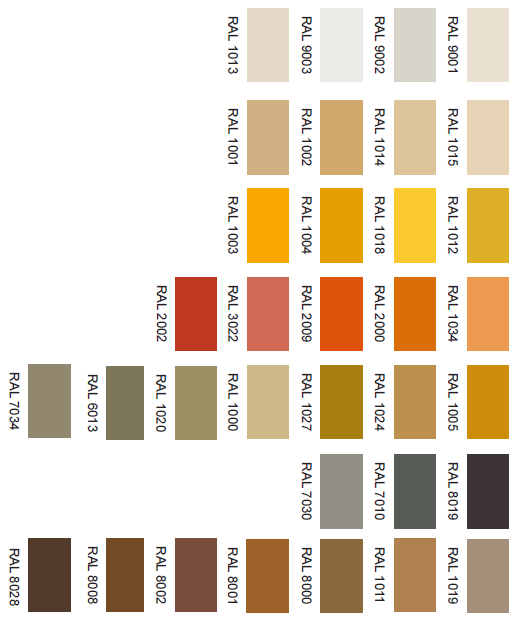


1. RAL colors <https://www.ralcolorchart.com>. Accessed January 3 2021.
