# Supplementary material for: The changing microRNA landscape by color and cloudiness: a cautionary tale for nipple aspirate fluid biomarker analysis
Source: Cell Oncol (Dordr). 2021 Oct 16;44(6):1339–49. doi: 10.1007/s13402-021-00641-w (PMC8648697; doi:10.1007/s13402-021-00641-w)
Supplement: Supplementary file 3 — (DOCX 15.7 KB) [file 13402_2021_641_MOESM3_ESM.docx]

**Supplementary Table 1.** Anthropomorphic characteristics of the study subjects from whom nipple aspirate fluid (NAF) samples were used to evaluate the effect of NAF color and cloudiness on miRNA expression levels, including intra-individual and inter-individual variation.

| **Characteristics** | n (%) or median (quartile 1 – quartile 3 (Q1-Q3)) |
| --- | --- |
| **DENSE-on cohort** | 154 |
| **Age** | 55 (52-58) |
| **BMI** | 24.7 (21.6-29.1)^a^ |
| **Mammographic density**  **___Volpara 1 or ‘a’**  **___Volpara 4 or ‘d’** | 73 (46.8%)  83 (53.2%) |
| **Age at menarche** | 13 (12-14)^b^ |
| **Parous**  ___**Age at first live birth**  **______<25**  **______25-29**  **______≥ 30** | 129 (87.2%)^c^  29 (22.5%)  57 (44.2%)  43 (33.3%) |
| **Postmenopausal** | 59 (39.6%)^d^ |
| **First degree relative with breast cancer** | 24 (19.9%)^e^ |
| a: 10 missings, b: 8 missings, c: 6 missings, d: 5 missings, e: 33 missings. NA: not applicable, LTR: lifetime risk. Valid percentages are shown i.e. without missings and not applicable data. | |

**Supplementary Table 2.** Frequencies of registered NAF appearances in the samples from the DENSE-on cohorts.

| **NAF color classes** | **Registered NAF color and cloudiness** | **Frequencies (n)** |
| --- | --- | --- |
| **Clear-colorless** | clear colorless | 53 |
| **Bloody** | clear orange | 14 |
|  | clear pink | 11 |
|  | clear bloody | 2 |
|  | clear white/orange | 1 |
|  | clear yellow/orange | 1 |
|  | cloudy pink | 4 |
|  | cloudy white/pink | 1 |
|  | cloudy bloody | 1 |
|  | cloudy orange | 1 |
| **Cloudy-white** | cloudy white | 30 |
| **Clear-yellow** | clear yellow | 20 |
| **Cloudy-yellow** | cloudy white/yellow | 4 |
|  | cloudy yellow | 15 |
| **Green** | clear green | 1 |
|  | clear green/blue | 1 |
|  | cloudy green | 4 |
|  | cloudy green/blue | 1 |
|  | cloudy yellow/green | 1 |
| **Brown** | cloudy brown | 4 |
|  | cloudy brown/yellow | 1 |
|  | cloudy brown/white | 2 |
